# Silver-catalysed azide–alkyne cycloaddition (AgAAC): assessing the mechanism by density functional theory calculations

**DOI:** 10.1098/rsos.160090

**Published:** 2016-09-14

**Authors:** Biswadip Banerji, K. Chandrasekhar, Sunil Kumar Killi, Sumit Kumar Pramanik, Pal Uttam, Sudeshna Sen, Nakul Chandra Maiti

**Affiliations:** 1 Organic and Medicinal Chemistry Division, CSIR-Indian Institute of Chemical Biology; 4, Raja S.C. Mullick Road, Kolkata, India; 2 Academy of Scientific and Innovative Research, CSIR-Indian Institute of Chemical Biology; 4, Raja S.C. Mullick Road, Kolkata, India; 3 Structural Biology and Bioinformatics Division, CSIR-Indian Institute of Chemical Biology; 4, Raja S.C. Mullick Road, Kolkata, India

**Keywords:** triazole, Ag-catalyst, click reaction, cycloaddition, DFT

## Abstract

‘Click reactions’ are the copper catalysed dipolar cycloaddition reaction of azides and alkynes to incorporate nitrogens into a cyclic hydrocarbon scaffold forming a triazole ring. Owing to its efficiency and versatility, this reaction and the products, triazole-containing heterocycles, have immense importance in medicinal chemistry. Copper is the only known catalyst to carry out this reaction, the mechanism of which remains unclear. We report here that the ‘click reactions’ can also be catalysed by silver halides in non-aqueous medium. It constitutes an alternative to the well-known CuAAC click reaction. The yield of the reaction varies on the type of counter ion present in the silver salt. This reaction exhibits significant features, such as high regioselectivity, mild reaction conditions, easy availability of substrates and reasonably good yields. In this communication, the findings of a new catalyst along with the effect of solvent and counter ions will help to decipher the still obscure mechanism of this important reaction.

## 1. Introduction

Reactions to produce small building blocks from selective components are synthetically highly demanding [1–3]. Copper (I)-catalysed Huisgen dipolar cycloaddition reaction of terminal alkynes with azides yields 1,4 and 1,5 triazoles [4,5]. It is the most convenient method for the synthesis of triazoles, which are widely used in chemistry, biology and materials science [6–9]. In such cycloaddition reactions (also known as click reactions), the products are obtained in very high yields with little or no by-product [10–12]. Click reactions can be performed under many conditions and are least affected by the nature of the other functional groups [13]. For these reasons, click chemistry has made a great impact in the pharmaceutical and synthetic world [14]. In the past years, considerable efforts have been made to enhance the efficiency and general applicability of this reaction [15,16]. All kinds of copper catalyst systems including the Cu/Cu_2_O nanoparticle catalyst systems have been developed to facilitate click chemistry and also to expand the substrate scope [17–20]. Nevertheless, the current transformation catalysed by silver salts and its mechanism have remained largely unexplored. Recently, Erick Cuevas [21] has described a process for the synthesis of 1,2,3-triazoles by using silver chloride and silver N-heterocyclic carbene complex. Abdul Aziz *et al*. [22] synthesized 1,4-disubstituted-1,2,3 triazoles by using AgN(CN)_2_ catalyst at room temperature. The use of silver (I) oxide nanoparticles and different silver (I) complexes was also reported for the synthesis of 1,4-disubstituted-1,2,3 triazoles [23–25]. In this work, we have demonstrated the catalytic activity of silver (I) in the Huisgen cycloaddition reaction of azides and alkynes and also a general computational investigation has been carried out to study the mechanisms of the silver-catalysed triazole formation reaction. It is noteworthy to mention here that the catalytic activity of Ag(I) species is remarkably controlled by its conjugate anion. This may be the reason for the better chemical yield over Cu(I) salt-catalysed reactions. Silver chloride salt in this reaction produces clean products with high yield. We have also explored the mechanism of this important transformation using quantum mechanical computations.

## 2. Results and discussion

**Scheme 1.**
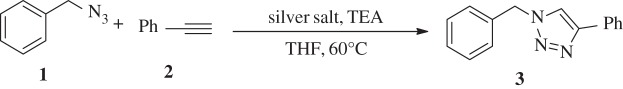
Silver catalysed click reaction.

In this study of the synthesis of silver-catalysed triazole compounds (**3a–l**), we screened several silver salts as catalysts for the click reaction (table 1). Here, we reported our findings on the synthesis of various silver-catalysed various triazole rings both in intermolecular and intramolecular fashion. The reaction was investigated in a series of control experiments. Accordingly, different acetylene compounds (**2**) were reacted with different azides (**1**), (1:1.2) in THF solvent, in the presence of different Ag(I) salts and the reaction mixture was heated at 60°C in the presence of approximately 5 equiv. of triethylamine, to get the desired triazole product, (**3**) Scheme 1. Among all the silver catalysts screened for this reaction (AgOAc, Ag_2_O, AgNO_3_, Ag_2_CO_3_, AgI and AgCl), AgCl produced the highest yield, 87% (table 1, entry 5). Yield in all the other cases was substantially lower, as shown in table 1. Without triethylamine, the reaction may proceed but it was extremely sluggish. Under the optimized reaction conditions (AgCl and TEA, table 1, entry 5), full conversion to the triazole product was achieved within 4–6 h at 60°C. With this optimized synthetic protocol, we further synthesized a small library of triazole compounds with different substituents as shown in table 2.

**Table 1.** Effects of different silver salts in triazole formation. All reactions are carried out with 0.5 mmol of **1** and 0.60 mmol of **2** in 4 ml THF.

entry	silver salt (20 mol%)	base (equiv.)	yield (%)
1	AgOAc	TEA (5)	44
2	Ag_2_O	TEA (5)	32
3	AgNO_3_	TEA (5)	13
4	Ag_2_CO_3_	TEA (5)	61
5	AgCl	TEA (5)	87
6	AgI	TEA (5)	45
7	AgCl	—	trace

**Table 2.** The list of different triazole compounds (**3a–l**) synthesized with the optimized condition.

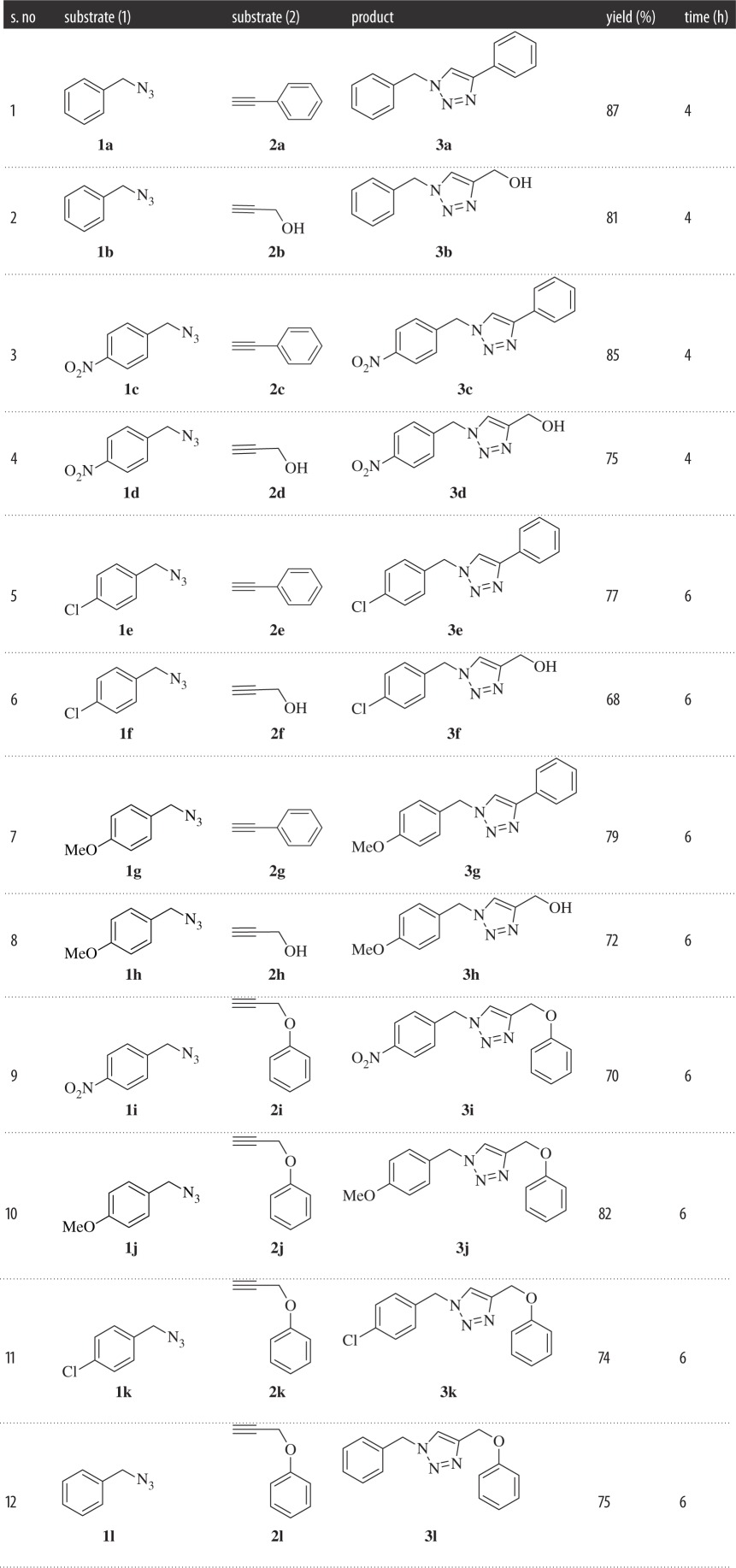

**Scheme 2.**
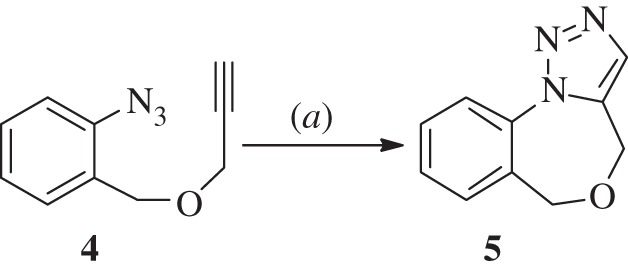
(*a*) AgCl (20 mol %), TEA (5 equiv.), THF, 60°C, 4 h.

The structures of all the synthesized triazole compounds (**3a–l**) were established spectroscopically by FTIR, ^1^H NMR, ^13^C NMR and HRMS (electronic supplementary material). After having the optimized condition in hand, we turned our attention to the one pot intramolecular triazole synthesis reaction. The four step reaction was initiated by converting the amine to the corresponding diazo compound followed by *in situ* displacement of the diazo group by azide resulting in compound **5**, scheme 2.

Thus, 1-azido-2-prop-2-ynyloxymethyl-benzene (**4**) undergoes intramolecular reaction to form 4H,6H-[1,2,3]triazolo[1,5-*a*][4,1]-benzoxazepine (**5**) in 75% yield under the same condition (scheme 2) [21].

## 3. Density functional theory calculation

Gaussian 09 Revision C.01 software was used for the quantum mechanical calculations. All the geometry optimizations were performed *in vaccuo* at density functional theory (DFT) level of theory using B3LYP/3–21G basis set for all the atoms. Molecules were drawn in GaussView 5. For theoretical calculations, the silver-catalysed 1,3 dipolar cycloaddition of methyl azide with propyne was studied. As proposed recently, for calculations we consider the involvement of the silver-centred acetylides, and the charge of the complexes was neutralized by adding chloride ions [26]. The initial guess of the transition state (TS) was obtained by scanning the N3–C4 and N1–C5 distances on a stable pre-reaction complex. All the other coordinates were relaxed during the scan. The TS was optimized using Berny algorithm (opt = ts) at the same level of theory. Molecular orbitals were calculated on the geometry optimized structures at the same level of theory. Coordinates for the optimized geometries are given in the electronic supplementary material. Relative energies were calculated with respect to the most stable pre-reaction complex. The energy values were converted to kilocalories per mole from Hartree per particle using the conversion factor of 627.509467.

**Figure 1.**
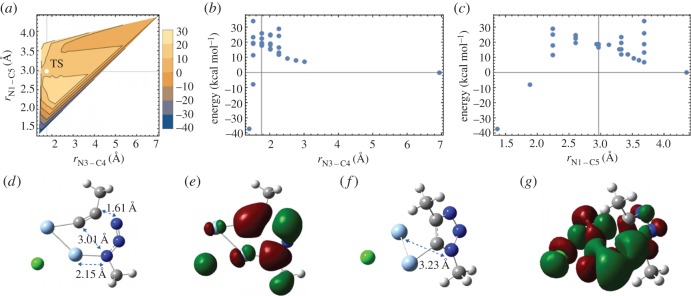
Quantum mechanical analysis of the silver-catalysed azide–alkyne cycloaddition. (*a*) Contour plot of the potential energy landscape for cycloaddition. Transition state (TS) is marked by a white dot. (*b*) Potential energy diagram for the N3–C4 bond formation. The vertical grid line indicates the TS bond length. (*c*) Potential energy diagram for N1–C5 bond formation. The vertical grid line indicates the TS bond length. (*d*) Transition state of the 1,4-disubstituted cycloaddition reaction involving a dinuclear silveracetylide. Colour key: H, white; C, grey; N, blue; Cl, green and Ag, cyan. Reaction coordinates are shown. (*e*) Molecular orbital (HOMO) of the transition state. (*f*) Reaction product 1,4-dimethyl-triazole attached to the metal centre. (*g*) HOMO of the product. A silver chloride is readily released from the product leaving (1,4-dimethyl-1H-1,2,3-triazol-5-yl) silver, the optimized geometry and molecular orbitals for which is given in the electronic supplementary material.

The Ag-catalysed reaction process has been modelled using quantum mechanical calculations. Figure 1*a* shows the potential energy landscape for the 1,4 disubstituted cycloaddition reaction. From the energy landscape, it appears that N3–C4 bond formation occurs at first, which then facilitates the N1–C5 bond formation. The saddle point in this potential energy landscape, which indicates the TS, is also highlighted in figure 1*a*. Figure 1*b*,*c* shows the reaction coordinates for N3–C4 and N1–C5 bond formations, respectively. The saddle point coordinates were used as initial guess for the TS optimization. Figure 1*d* shows the optimized geometry of the TS structure. Electron densities in the highest occupied molecular orbital (HOMO) at the TS are depicted in figure 1*e*. From this TS, the activation energy for the Ag-catalysed 1,4 disubstituted cycloaddition was computed to be 18.52 kcal mol^−1^. Optimized geometry of the product, i.e. after the N3–C4 and N1–C5 bond formation, is given in figure 1*f*. The Gibbs free energy for this two bond formation was found to be −37.51 kcal mol^−1^. Figure 1*g* shows HOMO of the reaction product.

It has been established that the click reactions are catalysed by dinuclear metal centres [16,27]. The detailed DFT analysis of the of copper-catalysed click reactions has also been reported previously in the literature [26]. According to the detailed DFT analysis by Cantillo *et al*. [26], the energy barriers for the uncatalysed azide–alkyne coupling in the absence of copper (I) species was approximately 36 kcal mol^−1^, whereas in the presence of a dinuclear copper centre the barrier leading to the 1,4-disubstituted triazole formation becomes approximately 16.0 kcal mol^−1^ which is comparable to the silver-catalysed reaction. However, the opposite regioisomer formation (1,5-approach) proceeds with a higher barrier, thus accounting for the observed regioselectivity [26]. Further, the comparison of the TS in silver-catalysed click reaction shows a very similar structure to that reported in the presence of the copper. In the presence of silver the N3–C4 and N1–C5 distances were calculated to be 1.61 Å and 3.01 Å, respectively (figure 1*d*), whereas in the presence of copper the N3–C4 and N1–C5 distances were 1.74 Å and 2.87 Å, respectively. N1–Cu distance was 2.01 Å, whereas the N1–Ag distance was computed to be 2.15 Å. In both the cases, the TS structures were planar in geometry.

## 4. Conclusion

We have reported a silver catalyst for click reactions illustrating the transformations which are experimentally simple, robust and reliable. We have successfully developed an AgAAC catalytic reaction method for the cycloaddition of different acetylenes with azide compounds. This reaction exhibited good general applicability and regioselectivity with a variety of acetylenes and azide compounds under mild conditions. We have also explored the mechanism of this reaction using DFT, which suggested the involvement of a dinuclear silver centre, which is also reported in copper-catalysed click reactions.

## 5. General procedure and characterization data

To an alkyne substrate (10 mmol) in THF (10 ml) was added AgCl (2 mmol), TEA (50 mmol) followed by azide substrate (12 mmol), and the reaction mixture was stirred vigorously at 60°C for 4 h. The reaction mixture was extracted with ethyl acetate, and washed with brine solution. After that the organic layer was separated, dried over sodium sulfate, filtered and evaporated under reduced pressure. The residue was finally purified by column chromatography (silica gel 100–200, ethyl acetate–hexane) to obtain the corresponding triazole compounds in 68–87% yield.

1-Benzyl-4-phenyl-1H-1,2,3-triazole (compound **3a**):


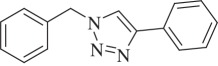


White solid; yield 87%; ^1^H NMR (600 MHz, CDCl_3_): *δ* (in ppm) 5.59 (2 H, s), 7.32–7.345 (3 H, m), 7.38–7.434 (5 H, m), 7.68 (1 H, s), 7.80–7.82 (2 H, m); ^13^C NMR (150 MHz, CDCl_3_): *δ* (in ppm) 147.79, 134.23, 130.07, 128.71, 128.36, 127.62, 127.61, 125.25, 119.04, 53.79; mass: [EI-HRMS] (C_15_H_13_N_3_) calc. 235.1109 Da, found: 235.1089 Da; FTIR (KBr, *ν*_max_, cm^−1^): 3500, 3139, 3039, 1608, 1458, 1353, 1215, 1070, 972, 808, 764, 720, 695.

(1-Benzyl-1H-1,2,3-triazol-4-yl)methanol (compound **3b**):


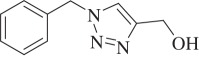


White solid; yield 81%; ^1^H NMR (600 MHz, CDCl_3_): *δ* (in ppm) 2.43 (1 H, s), 4.78 (2 H, d, *J* = 6), 5.53 (2 H, s), 7.28–7.302 (2 H, m), 7.37–7.41 (3 H, m), 7.46 (1 H, s); ^13^C NMR (150 MHz, CDCl_3_): *δ* (in ppm) 147.54, 134.01, 128.71, 128.39, 127.69, 121.09, 56.19, 53.77; mass: [EI-HRMS] (C_10_H_11_N_3_O) calc. 189.0902 Da, found: 189.0903 Da; FTIR (KBr, *ν*_max_, cm^−1^): 3352, 3141, 2927, 2861, 1607, 1553, 1496, 1335, 1221, 1127, 1046, 797, 722.

1-(4-Nitrobenzyl)-4-phenyl-1H-1,2,3-triazole (compound **3c**):


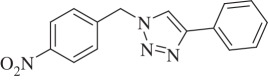


White solid; yield 85%; ^1^H NMR (600 MHz, CDCl_3_): *δ* (in ppm) 5.71 (2 H, s), 7.345–7.375 (1 H, m), 7.42–7.47 (4 H, m), 7.77 (1 H, s), 7.81–7.84 (2 H, m), 8.24–8.26 (2 H, m); ^13^C NMR (150 MHz, CDCl_3_): *δ* (in ppm) 148.28, 147.64, 141.30, 129.63, 128.47, 128.10, 128.06, 125.29, 123.9, 119.24, 52.73; mass: [EI-HRMS] (C_15_H_12_N_4_O_2_) calc. 280.0960 Da, found: 280.0967 Da; FTIR (KBr, *ν*_max_, cm^−1^): 3124, 3084, 1706, 1606, 1517, 1348, 1215, 1071, 1044, 866, 762, 692.

(1-(4-Nitrobenzyl)-1H-1,2,3-triazol-4-yl)methanol (compound **3d**):


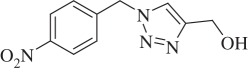


White solid; yield 75%; ^1^H NMR (600 MHz, CDCl_3_): *δ* (in ppm) 2.43 (1 H, s), 4.83 (2 H, s), 5.66 (2 H, s), 7.425 (2 H, d, *J* = 6), 7.56 (1 H, s), 8.245 (2 H, d, *J* = 6); ^13^C NMR (150 MHz, CDCl_3_): *δ* (in ppm) 147.66, 141.05, 128.16, 123.90, 121.44, 56.13, 52.72; mass: [EI-HRMS] (C_10_H_10_N_4_O_3_) calc. 234.0753 Da, found: 234.0744 Da; FTIR (KBr, *ν*_max_, cm^−1^): 3263, 3113, 1608, 1536, 1468, 1349, 1227, 1126, 1012, 854, 797, 729, 678.

1-(4-Chlorobenzyl)-4-phenyl-1H-1,2,3-triazole (compound **3e**):


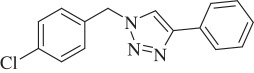


White solid; yield 75%; ^1^H NMR (300 MHz, CDCl_3_): *δ* (in ppm) 5.54 (2 H, s), 7.21–7.26 (2 H, m), 7.3–7.45 (5 H, m), 7.67 (1 H, s,), 7.77–7.82 (2 H, m); ^13^C NMR (75 MHz, CDCl_3_): *δ* (in ppm) 134.77, 133.15, 130.30, 129.69, 129.32, 128.80, 128.24, 126.04, 125.65, 119.44, 53.41; mass: [EI-HRMS] (C_15_H_12_ClN_3_) calc. 269.0720 Da, found: 271.0702 Da; FTIR (KBr, *ν*_max_, cm^−1^): 3447, 3082, 1488, 1462, 1351, 1217, 1083, 1015, 808, 764.

(1-(4-Chlorobenzyl)-1H-1,2,3-triazol-4-yl)methanol (compound **3f**):


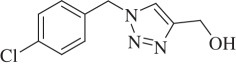


White solid; yield 68%; ^1^H NMR (600 MHz, CDCl_3_): *δ* (in ppm) 4.75 (2 H, s), 5.5 (2 H, s), 7.21 (2 H, d, *J* = 6), 7.34 (2 H, *J* = 12), 7.47 (1 H, s,); ^13^C NMR (150 MHz, CDCl_3_): *δ* (in ppm) 147.84, 134.38, 132.51, 128.97, 128.87, 128.64, 128.61, 121.25, 55.78, 52.97; mass: [EI-HRMS] (C_10_H_10_ClN_3_O) calc. 223.0512 Da, found: 223.0491 Da; FTIR (KBr, *ν*_max_, cm^−1^): 3263, 3110, 2923, 2853, 1596, 1489, 1439, 1291, 1230, 1086, 1022, 858, 777, 661.

1-(4-Methoxybenzyl)-4-phenyl-1H-1,2,3-triazole (compound **3g**):


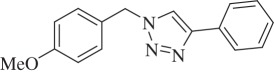


White solid; yield 79%; ^1^H NMR (600 MHz, CDCl_3_): *δ* (in ppm) 3.82 (3 H, s), 5.52 (2 H, s), 6.91–6.94 (2 H, m), 7.27–7.29 (2 H, m), 7.30–7.33 (1 H, m), 7.40 (2 H, t, *J* = 6), 7.63 (1 H, s), 7.78–7.80 (2 H, m); ^13^C NMR (150 MHz, CDCl_3_): *δ* (in ppm) 159.50, 147.68, 130.07, 129.23, 128.34, 127.69,126.13, 125.22, 118.83, 114.06, 54.90, 53.35; mass: [EI-HRMS] (C_16_H_15_N_3_O) calc. 265.1215 Da, found: 265.1223 Da; FTIR (KBr, *ν*_max_, cm^−1^): 3449, 3123, 2932, 2839, 1610, 1513, 1459, 1301, 1247, 1073, 1026, 828, 763, 694.

(1-(4-Methoxybenzyl)-1H-1,2,3-triazol-4-yl)methanol (compound-**3 h**):


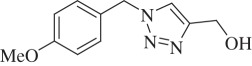


White solid; yield 72%; ^1^H NMR (300 MHz, CDCl_3_): *δ* (in ppm) 3.04 (1 H, s), 3.79 (3 H, s), 4.73 (2 H, s), 5.43 (2 H, s,), 6.88 (2 H, d, *J* = 6), 7.22 (2 H, d, *J* = 9), 7.41 (1 H, s); ^13^C NMR (75 MHz, CDCl_3_): *δ* (in ppm) 158.87, 148.09, 129.70, 126.43, 121.52, 114.52, 56.14, 55.32, 53.68; mass: [ESI-HRMS] (C_11_H_13_N_3_O_2_) (M + Na^+^) calc. 242.0905 Da, found: 242.0819 Da; FTIR (KBr, *ν*_max_, cm^−1^): 3288, 3115, 2691, 2838, 1609,1513, 1460, 1247, 1034, 846, 785, 644.

1-(4-Nitrobenzyl)-4-(phenoxymethyl)-1H-1,2,3-triazole (compound **3i**):


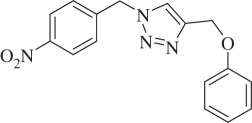


White solid; yield 70%; ^1^H NMR (600 MHz, CDCl_3_): *δ* (in ppm) 5.24 (2 H, s), 5.67 (2 H, s), 6.96–7.00 (3 H, m), 7.31 (2 H, t, *J* = 6), 7.42 (2 H, d, *J* = 12), 7.63 (1 H, s), 8.25 (2 H, t, *J* = 6); ^13^C NMR (150 MHz, CDCl_3_): *δ* (in ppm) 157.57, 147.66, 144.92, 141.02, 129.14, 128.14, 128.04, 127.81, 123.90, 123.64, 123.54, 122.34, 120.96, 114.27, 61.49, 52.74; mass: [EI-HRMS] (C_16_H_14_N_4_O_3_) calc. 310.1066 Da, found: 310.1055 Da; FTIR (KBr, *ν*_max_, cm^−1^): 3291, 2925, 1646, 1599, 1515, 1346, 1240, 1008, 829, 733.

1-(4-Methoxybenzyl)-4-(phenoxymethyl)-1H-1,2,3-triazole (compound **3j**):


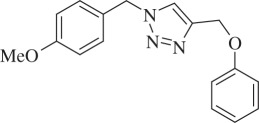


White solid; yield 82%; ^1^H NMR (300 MHz, CDCl_3_): *δ* (in ppm) 3.81 (3 H, s), 5.18 (2 H, s), 5.46 (2 H, s), 6.88 (1 H, s), 6.91 (1 H, s), 6.94–6.99 (3 H, m), 7.22 (1 H, s), 7.26 (2 H, d, *J* = 6), 7.31 (1 H, s), 7.49 (1 H, s); ^13^C NMR (75 MHz, CDCl_3_): *δ* (in ppm) 159.91, 158.15, 144.52, 129.70, 129.48, 126.36, 122.32, 121.18, 114.69, 114.45, 61.97, 55.30, 53.76; mass: [EI-HRMS] (C_17_H_17_N_3_O_2_) calc. 295.1321 Da, found: 295.1326 Da; FTIR (KBr, *ν*_max_, cm^−1^): 3075, 1605, 1513, 1245, 1176, 1033, 841, 754.

1-(4-Chlorobenzyl)-4-(phenoxymethyl)-1H-1,2,3-triazole (compound **3k**):


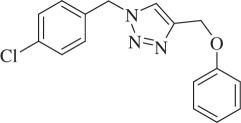


White solid; yield 74%; ^1^H NMR (600 MHz, CDCl_3_): *δ* (in ppm) 5.21 (2 H, s), 5.51 (2 H, s), 6.98 (3 H, d, *J* = 12), 7.22 (2 H, d, *J* = 6), 7.285–7.315 (2 H, m), 7.36 (2 H, d, *J* = 6), 7.55 (1 H, s); ^13^C NMR (150 MHz, CDCl_3_): *δ* (in ppm) 157.67, 144.49, 134.44, 132.51, 129.10, 128.97, 128.92, 122.06, 120.86, 114.30, 61.55, 53.04; mass: [EI-HRMS] (C_16_H_14_ClN_3_O) calc. 299.0825 Da, found: 299.0823 Da; FTIR (KBr, *ν*_max_, cm^−1^): 3138, 3100, 2928, 1593, 1492, 1293, 1224, 1087, 1008, 851, 751.

1-Benzyl-4-(phenoxymethyl)-1H-1,2,3-triazole (compound **3l**):


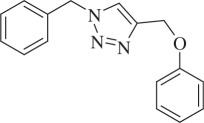


White solid; yield 75%; ^1^H NMR (600 MHz, CDCl_3_): *δ* (in ppm) 5.21 (2 H, s), 5.55 (2 H, s), 6.96–7 (3 H, m), 7.28–7.31 (4 H, m), 7.39 (3 H, t, *J* = 6), 7.54 (1 H, s); ^13^C NMR (150 MHz, CDCl_3_): *δ* (in ppm) 157.71, 144.26, 133.98, 129.06, 128.69, 128.36, 127.67, 122.08, 120.79, 114.29, 61.59, 53.81; mass: [EI-HRMS] (C_16_H_15_N_3_O) calc. 265.1215 Da, found: 265.1206 Da; FTIR (KBr, *ν*_max_, cm^−1^): 3131, 2923, 2855, 1738, 1593, 1493, 1221, 756.

## Supplementary Material

In supplementary information the detailed DFT calculations and NMR spectra of the final compounds are provided

## Data accessibility

The detailed DFT calculations and NMR spectra of the final compounds are provided in the electronic supplementary material.
