# Supplementary material for: Silver-catalysed azide–alkyne cycloaddition (AgAAC): assessing the mechanism by density functional theory calculations
Source: R Soc Open Sci. 2016 Sep 14;3(9):160090. doi: 10.1098/rsos.160090 (PMC5043300; doi:10.1098/rsos.160090)
Supplement: In supplementary information the detailed DFT calculations and NMR spectra of the final compounds are provided [file rsos160090supp1.docx]

**Data Availability**

**Silver-Catalyzed Azide–Alkyne Cycloaddition (AgAAC): Assessing the Mechanism by DFT Calculations**

**Biswadip Banerji,^*a,b^ K Chandrasekhar,^a^ Sunil Kumar Killi,^a^ Sumit Kumar Pramanik,^a^  Uttam Pal,^c^ Sudeshna Sen^c^ and Nakul Chandra Maiti^c^**

[a] Organic and Medicinal Chemistry Division, CSIR-Indian Institute of Chemical Biology; 4, Raja S.C. Mullick Road, Kolkata, India E-mail: biswadip.banerji@gmail.com

[b] Academy of Scientific and Innovative Research,CSIR-Indian institute of Chemical biology; 4 Raja S.C. MullickRoad,Kolkata,India.

[c] Structural Biology and Bioinformatics Division, CSIR-Indian Institute of Chemical Biology; 4, Raja S.C. Mullick Road, Kolkata, India.

**Theoretical study with *DFT* methods**

*Silver acetylide*


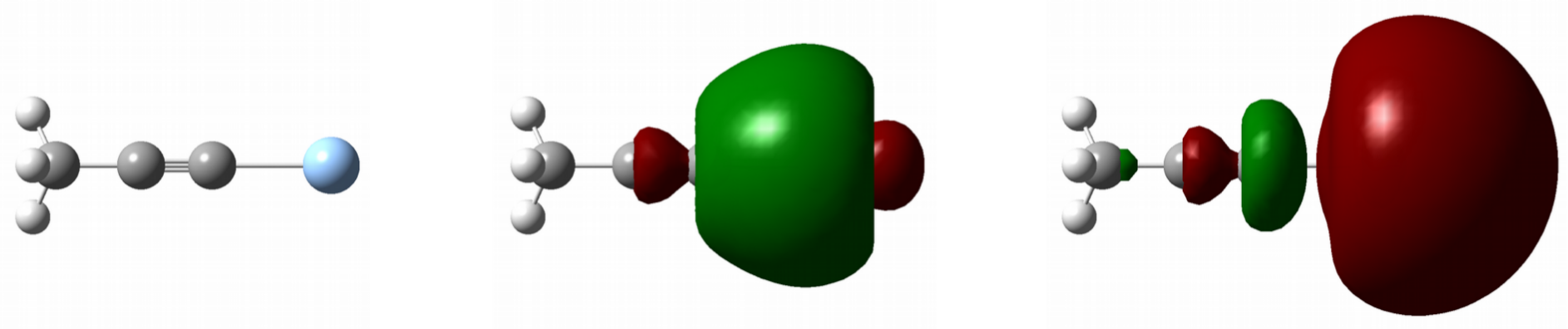


HOMO (-0.255465695 a.u.) LUMO (-0.148914449 a.u.)

E(RB3LYP) = -5291.72036157 a.u.

Standard orientation:

---------------------------------------------------------------------

Center Atomic Atomic Coordinates (Angstroms)

Number Number Type X Y Z

---------------------------------------------------------------------

1 6 0 0.000000 0.000000 -3.620560

2 1 0 0.000000 1.024061 -4.015933

3 1 0 0.886863 -0.512031 -4.015933

4 1 0 -0.886863 -0.512031 -4.015933

5 6 0 0.000000 0.000000 -2.155944

6 6 0 0.000000 0.000000 -0.934278

7 47 0 0.000000 0.000000 1.113032

---------------------------------------------------------------------

*Methyl azide·AgCl*


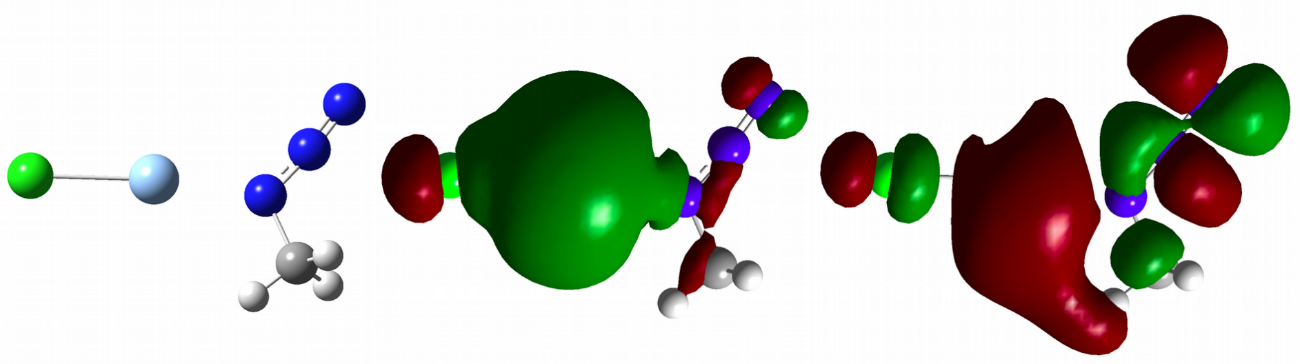


HOMO (-0.246905236 a.u.) LUMO (-0.128369087 a.u.)

E(RB3LYP) = -5837.33239394 a.u.

Standard orientation:

---------------------------------------------------------------------

Center Atomic Atomic Coordinates (Angstroms)

Number Number Type X Y Z

---------------------------------------------------------------------

1 7 0 3.203755 -1.564375 -0.000458

2 7 0 2.524172 -0.638214 -0.000085

3 7 0 1.670141 0.305963 0.000482

4 47 0 -0.497333 -0.027117 0.000256

5 6 0 2.282182 1.693798 -0.000400

6 1 0 2.883415 1.846226 -0.900768

7 1 0 1.436957 2.379447 0.000435

8 1 0 2.885444 1.846537 0.898555

9 17 0 -2.900632 -0.099065 -0.000437

---------------------------------------------------------------------

*Stable pre-reaction complex*


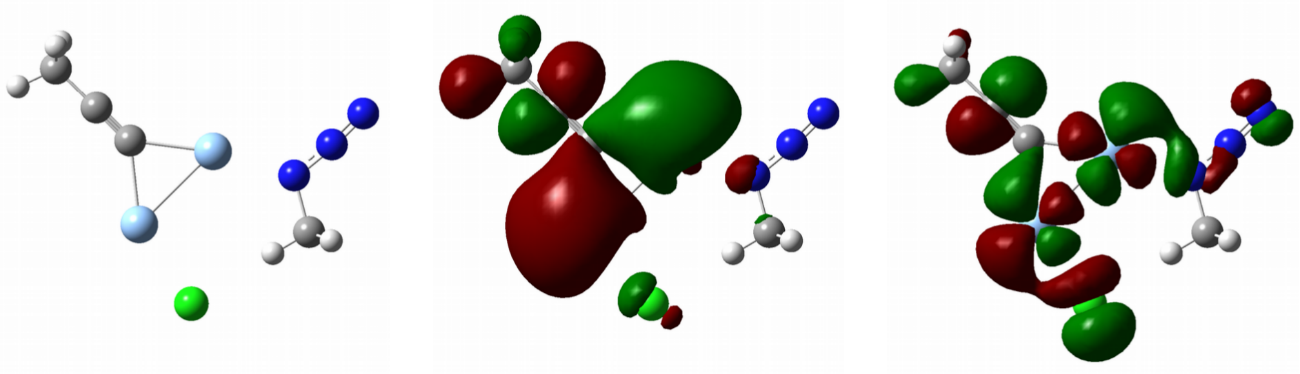


HOMO (-0.267116591 a.u.) LUMO (-0.166296004 a.u.)

E(RB3LYP) = -11129.1399029 a.u.

Standard orientation:

---------------------------------------------------------------------

Center Atomic Atomic Coordinates (Angstroms)

Number Number Type X Y Z

---------------------------------------------------------------------

1 6 0 3.580118 3.050417 -0.001163

2 1 0 3.455191 3.749906 0.834569

3 1 0 4.566236 2.579954 0.100255

4 1 0 3.575715 3.625440 -0.935199

5 6 0 2.525450 2.035879 -0.000158

6 6 0 1.648520 1.173977 0.000722

7 47 0 1.411837 -1.014401 -0.000007

8 47 0 -0.506684 0.893292 0.000408

9 7 0 -2.616149 0.264524 -0.000298

10 7 0 -3.597442 1.071434 -0.000486

11 7 0 -4.414430 1.879644 -0.000780

12 6 0 -3.004368 -1.212335 0.000267

13 1 0 -2.064035 -1.770607 -0.000090

14 1 0 -3.578914 -1.441735 0.901544

15 1 0 -3.579901 -1.442215 -0.900251

16 17 0 0.057726 -3.082675 -0.000394

---------------------------------------------------------------------

*Transition state*


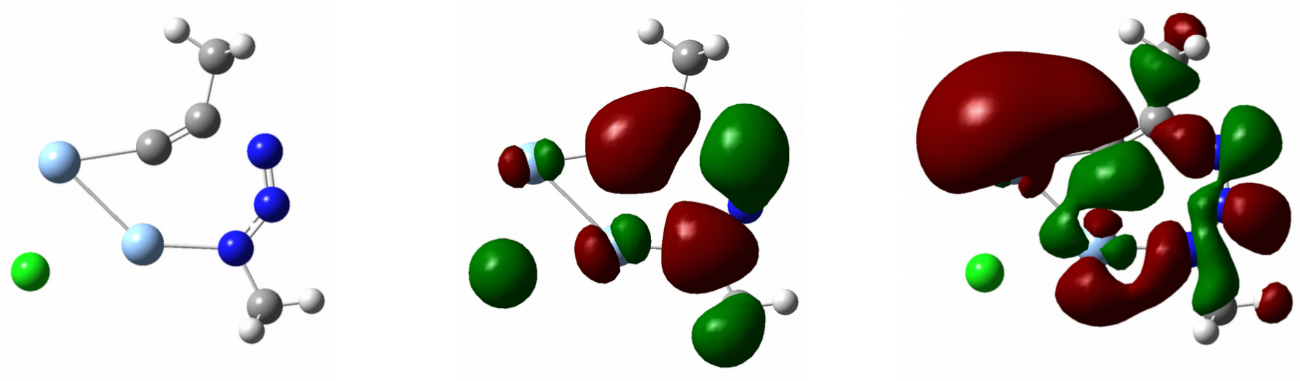


HOMO (-0.231242082 a.u.) LUMO (-0.107613601 a.u.)

E(RB3LYP) = -11129.1103828 a.u.

Standard orientation:

---------------------------------------------------------------------

Center Atomic Atomic Coordinates (Angstroms)

Number Number Type X Y Z

---------------------------------------------------------------------

1 6 0 1.871241 3.373583 -0.000076

2 1 0 2.470801 3.607311 -0.885864

3 1 0 0.960914 3.975536 -0.000045

4 1 0 2.470889 3.607349 0.885642

5 6 0 1.555014 1.897702 -0.000030

6 6 0 0.426265 1.278581 0.000036

7 47 0 -1.755850 0.934295 0.000052

8 47 0 0.171983 -0.964275 0.000023

9 7 0 2.321720 -1.057186 -0.000035

10 7 0 3.154634 -0.043678 -0.000055

11 7 0 2.996945 1.186103 -0.000084

12 6 0 2.990202 -2.398207 0.000100

13 1 0 2.689359 -2.955450 0.892677

14 1 0 2.688829 -2.955850 -0.892045

15 1 0 4.073792 -2.268905 -0.000251

16 17 0 -2.428368 -1.594562 -0.000151

---------------------------------------------------------------------

*1,4 di substituted product*


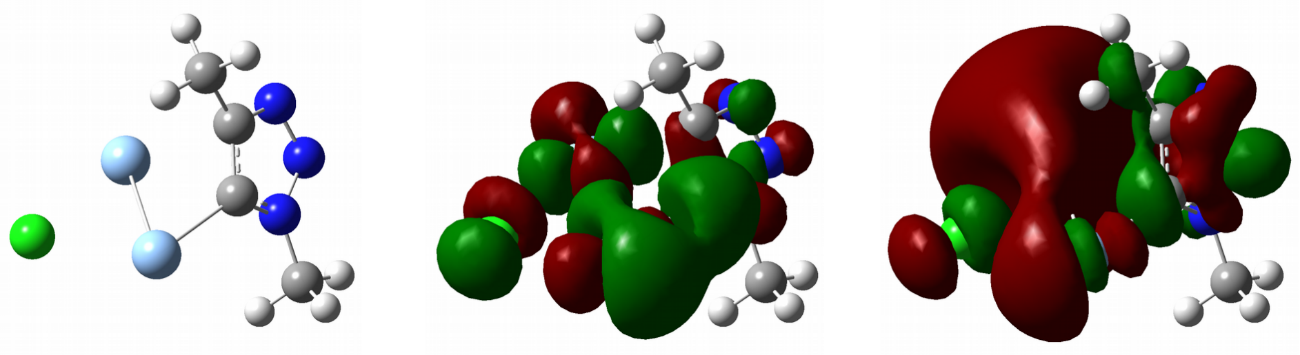


HOMO (-0.24226 a.u.) LUMO (-0.07903 a.u.)

E(RB3LYP) = -11129.1996762 a.u.

Standard orientation:

---------------------------------------------------------------------

Center Atomic Atomic Coordinates (Angstroms)

Number Number Type X Y Z

---------------------------------------------------------------------

1 6 0 -1.126063 -1.339251 2.161776

2 1 0 -1.242133 -2.421716 2.047100

3 1 0 -0.074048 -1.109039 2.362486

4 1 0 -1.706253 -1.036003 3.041564

5 6 0 -1.610866 -0.602351 0.936758

6 6 0 -1.147509 0.650430 0.430284

7 47 0 0.965452 1.263360 0.494012

8 47 0 0.233872 -1.264070 -0.511056

9 7 0 -2.104817 0.965776 -0.497807

10 7 0 -3.146520 -0.026459 -0.578531

11 7 0 -2.812495 -0.963552 0.309140

12 6 0 -2.187595 2.124951 -1.391269

13 1 0 -1.365576 2.111595 -2.113673

14 1 0 -3.140299 2.050797 -1.917072

15 1 0 -2.152297 3.049164 -0.810200

16 17 0 2.717141 -0.437908 -0.544943

---------------------------------------------------------------------

**(***1,4-dimethyl-1H-1,2,3-triazol-5-yl)silver*


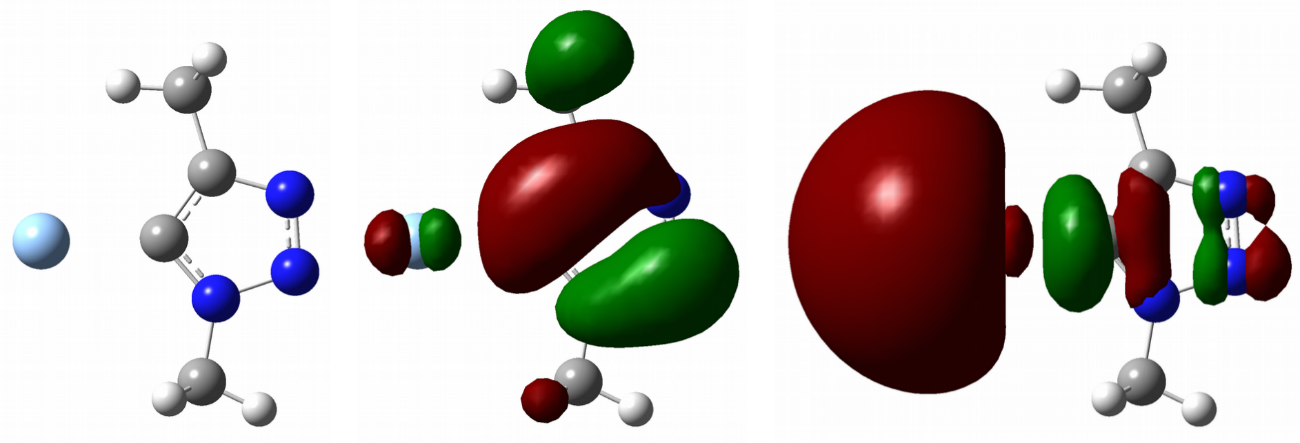


HOMO (-0.2104436480 a.u.) LUMO (-0.0973455214 a.u.)

E(RB3LYP) = -5494.76236536 a.u.

Standard orientation:

---------------------------------------------------------------------

Center Atomic Atomic Coordinates (Angstroms)

Number Number Type X Y Z

---------------------------------------------------------------------

1 6 0 0.884102 2.579379 0.000112

2 1 0 1.262171 3.103331 -0.885352

3 1 0 -0.209987 2.640853 0.000362

4 1 0 1.262588 3.103281 0.885429

5 6 0 1.304066 1.141531 -0.000018

6 6 0 0.489732 0.007893 0.000169

7 47 0 -1.608364 -0.041246 -0.000030

8 7 0 1.373055 -1.038101 0.000109

9 7 0 2.724452 -0.572891 -0.000119

10 7 0 2.636951 0.768270 -0.000172

11 6 0 1.133223 -2.478352 0.000067

12 1 0 0.579819 -2.778245 -0.895121

13 1 0 2.113836 -2.956684 0.001876

14 1 0 0.576744 -2.777636 0.893532

---------------------------------------------------------------------


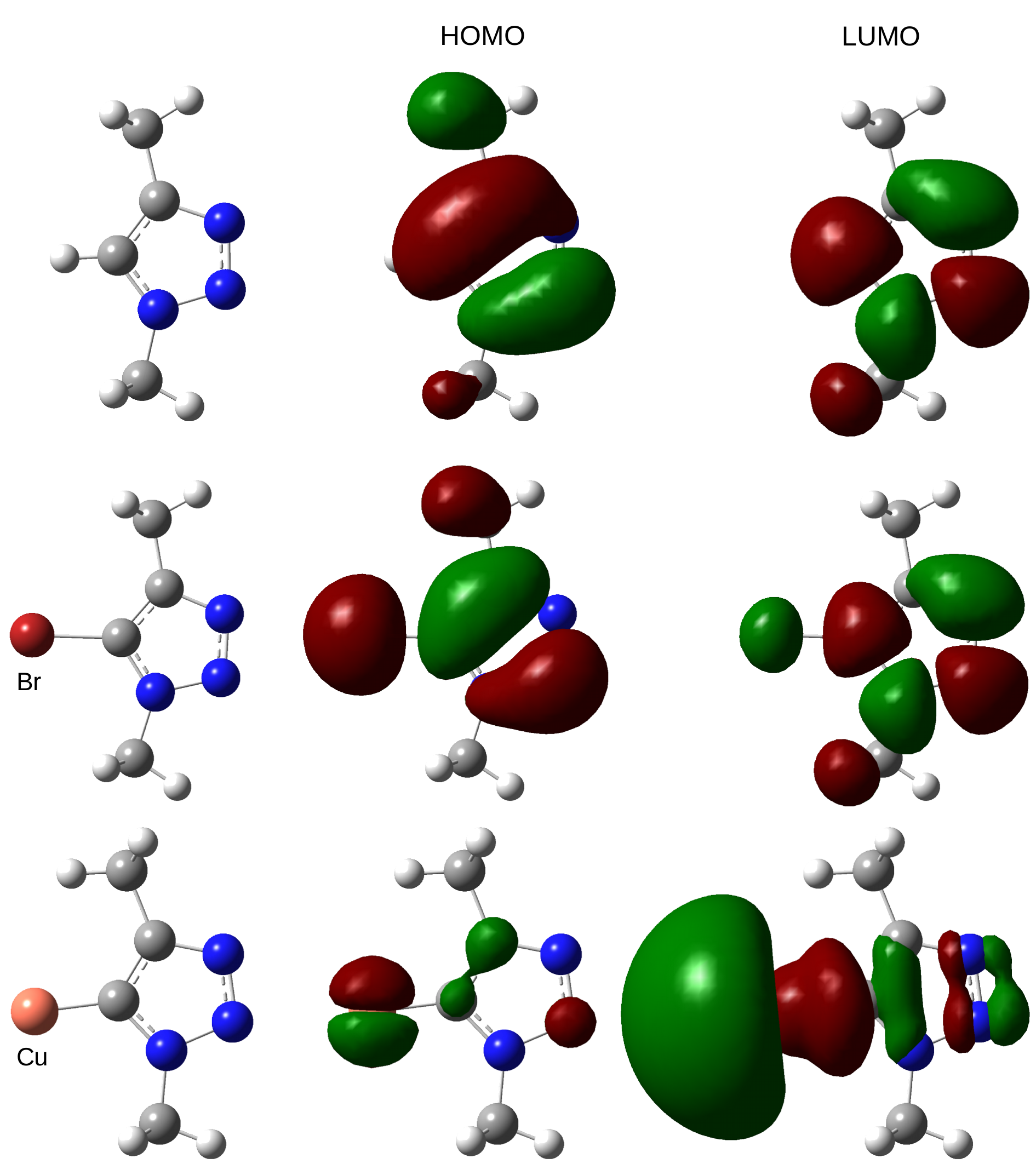


**Figure S1** HOMO/LUMO orbitals of 1,4-dimethyl-1H-1,2,3-triazole; 5-bromo-1,4-dimethyl-1H-1,2,3-triazole and (1,4-dimethyl-1H-1,2,3-triazol-5-yl)copper.

**Some selected spectra:**

**1-benzyl-4-phenyl-1H-1,2,3-triazole (compound-3a) 1H NMR:**

**
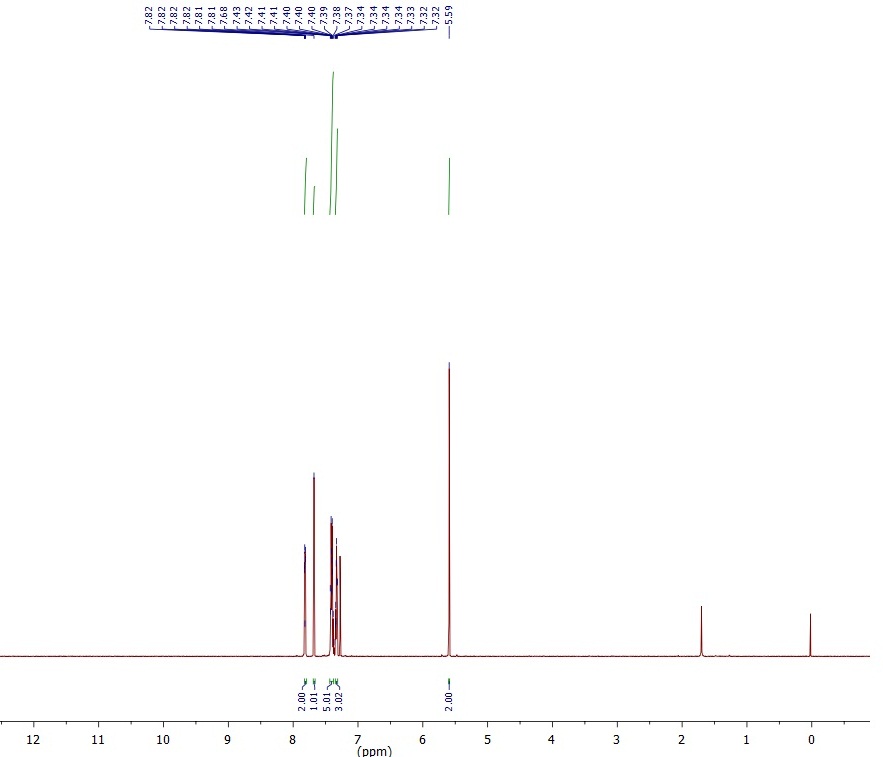
**

**1-benzyl-4-phenyl-1H-1,2,3-triazole (compound-3a) 13C NMR:**

**
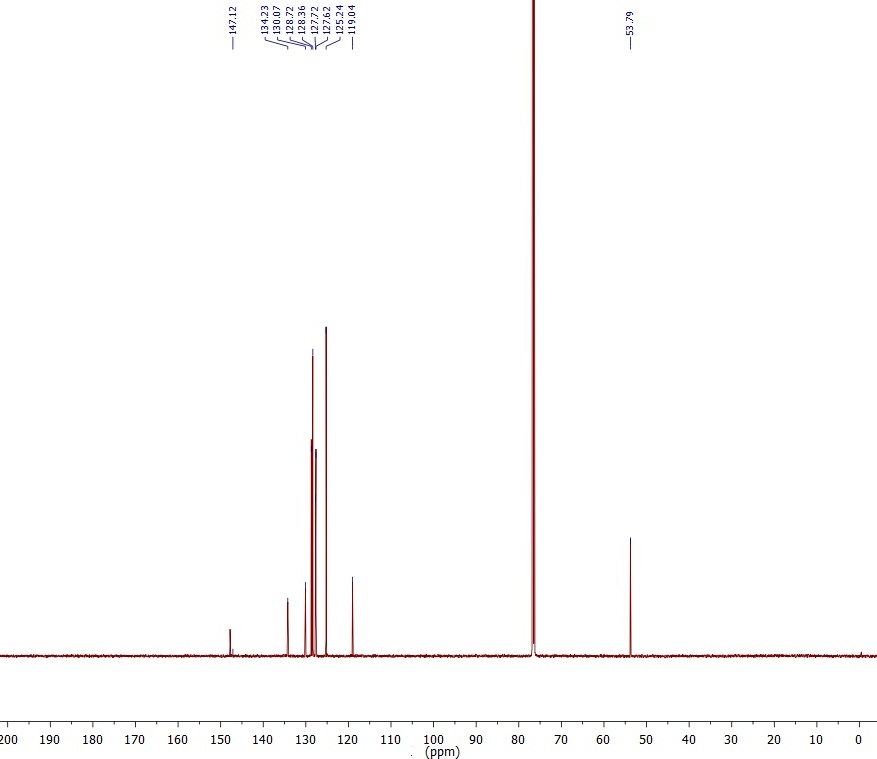
**

**(1-benzyl-1H-1,2,3-triazol-4-yl)methanol (compound-3b) 1H NMR:**

**
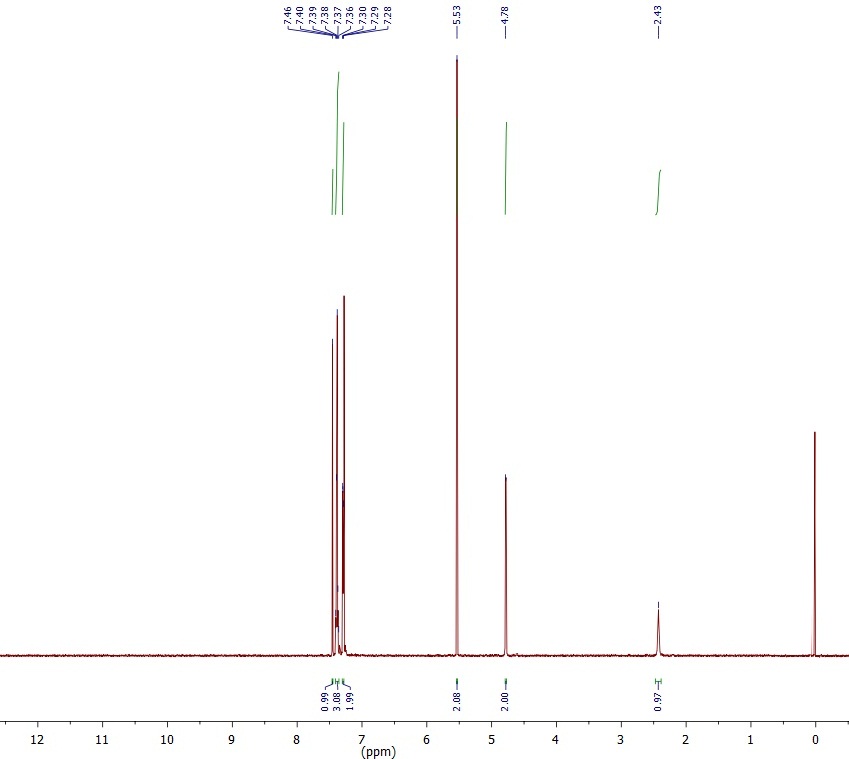
**

**(1-benzyl-1H-1,2,3-triazol-4-yl)methanol (compound-3b) 13C NMR:**

**
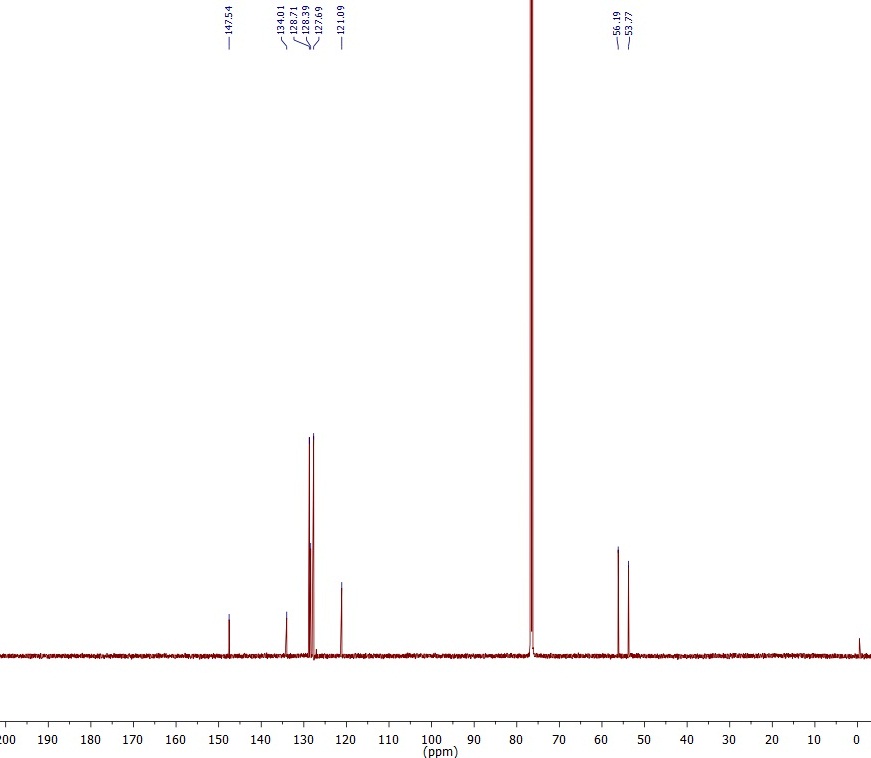
**

**1-(4-nitrobenzyl)-4-phenyl-1H-1,2,3-triazole (compound-3c) 1H NMR:
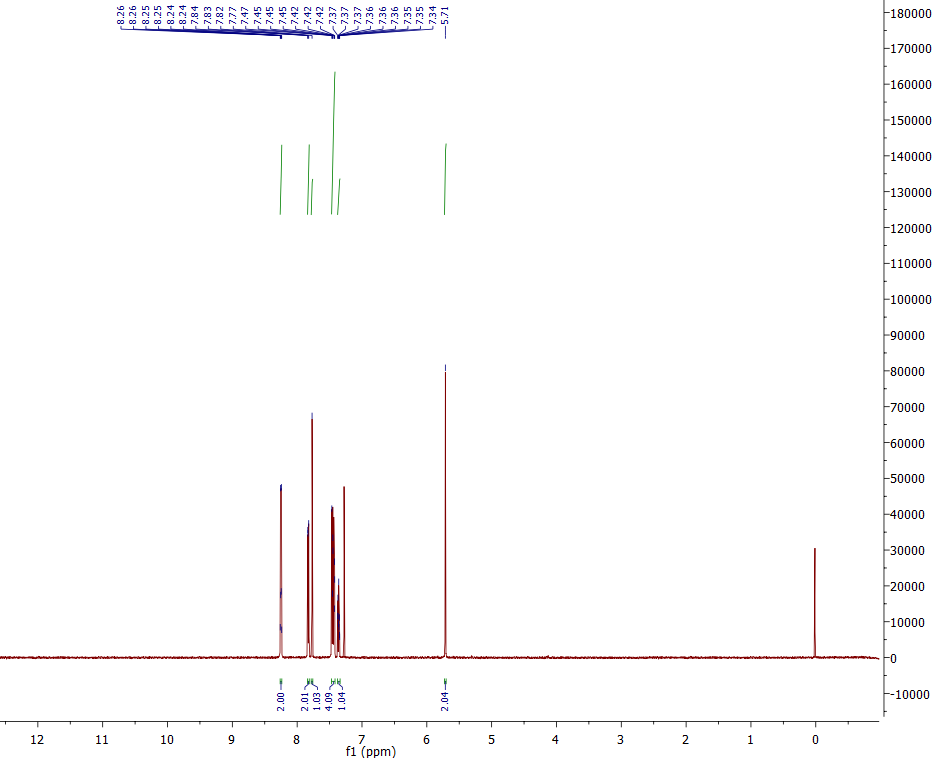
**

**1-(4-nitrobenzyl)-4-phenyl-1H-1,2,3-triazole (compound-3c) 13C NMR:
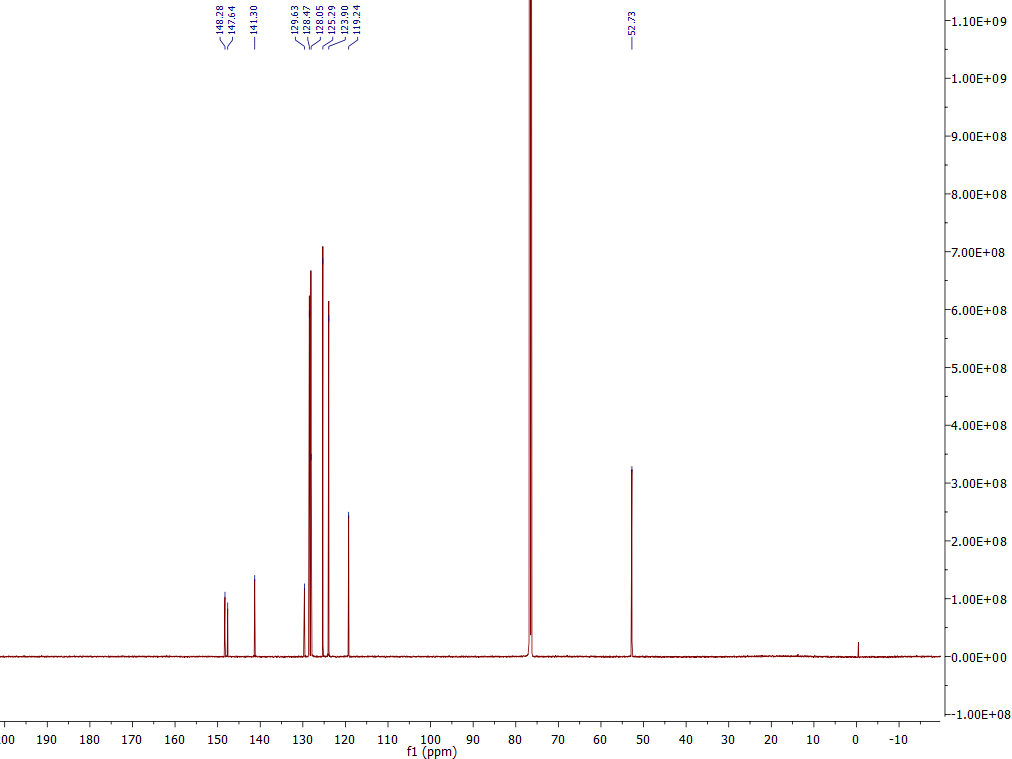
**

**(1-(4-nitrobenzyl)-1H-1,2,3-triazol-4-yl)methanol (compound-3d)1H NMR:**

**
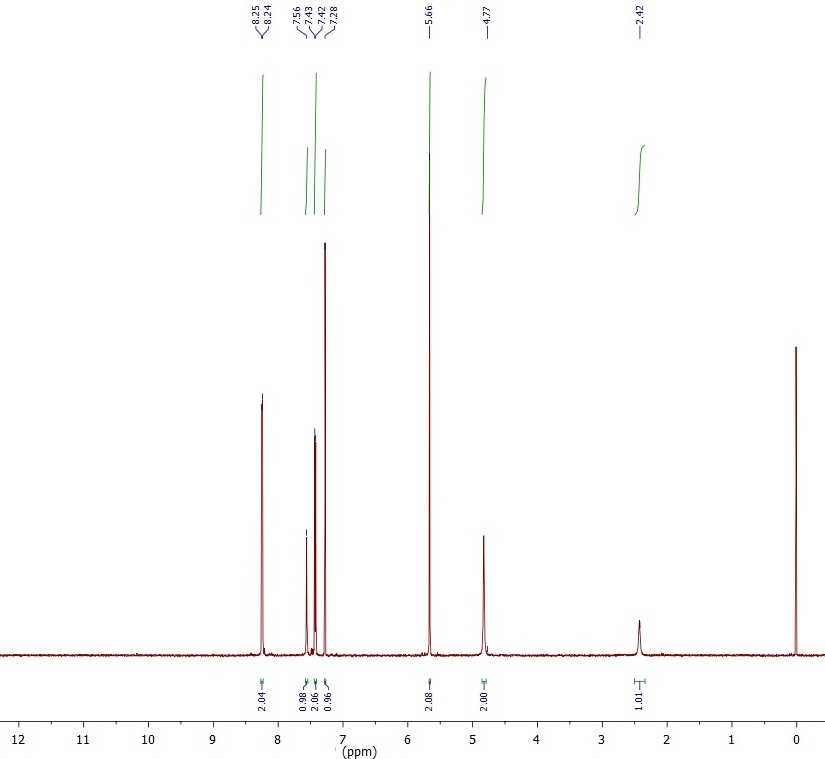
**

**(1-(4-nitrobenzyl)-1H-1,2,3-triazol-4-yl)methanol (compound-3d)13C NMR:**

**
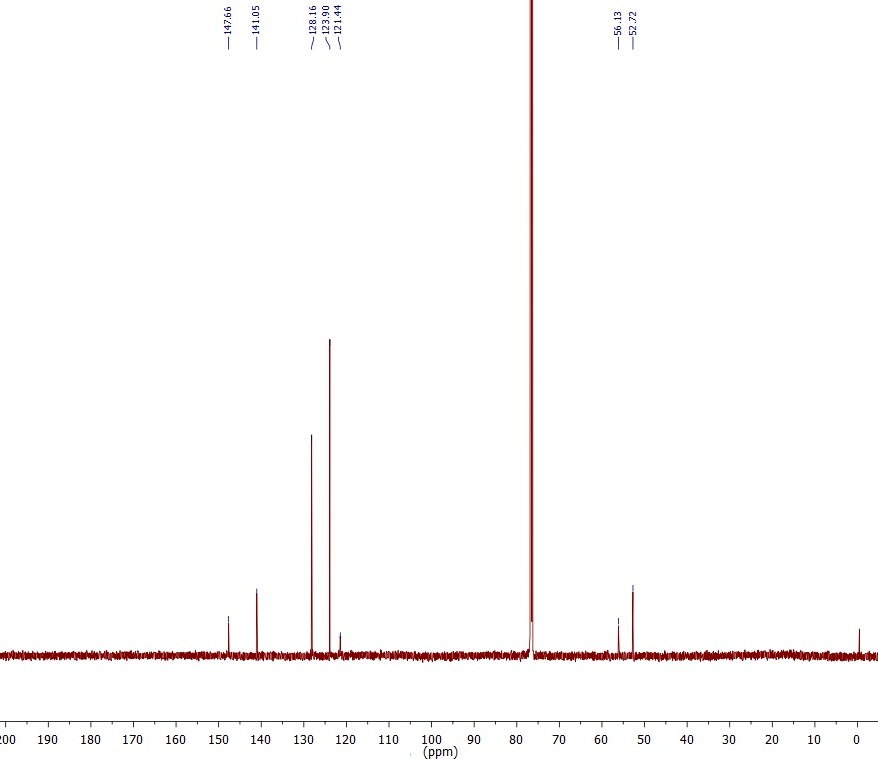
**

**1-(4-chlorobenzyl)-4-phenyl-1H-1,2,3-triazole (compound-3e) 1H NMR:**

**
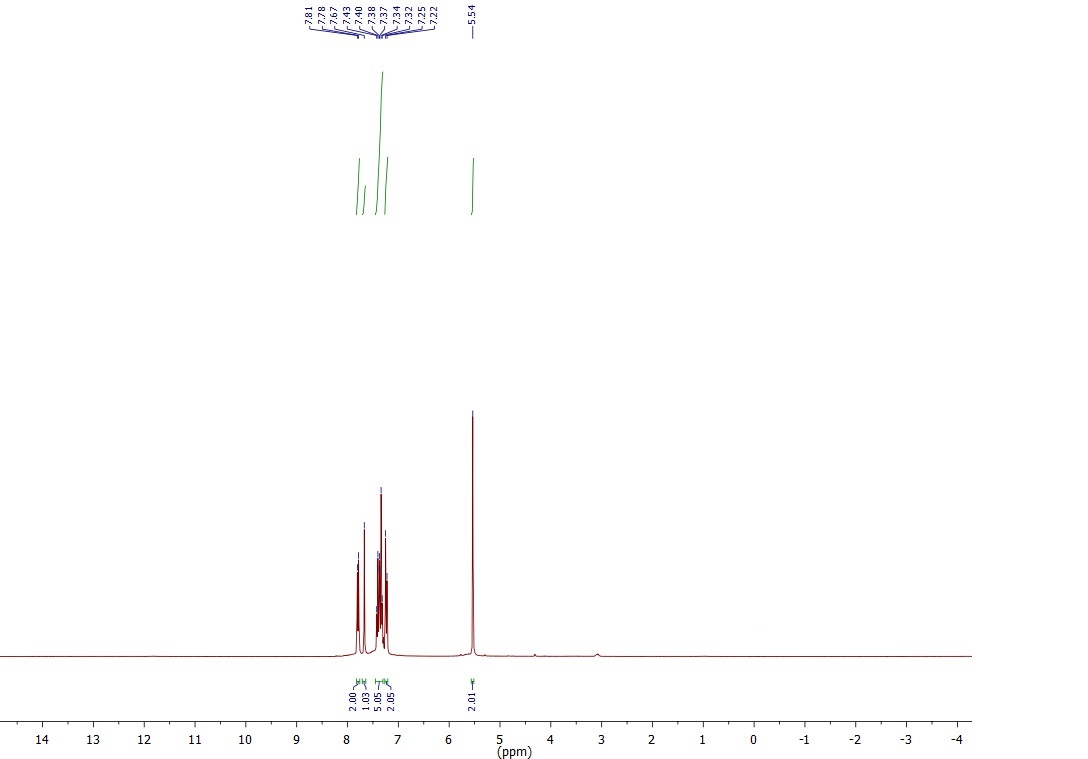
**

**1-(4-chlorobenzyl)-4-phenyl-1H-1,2,3-triazole (compound-3e) 13C NMR:**

**
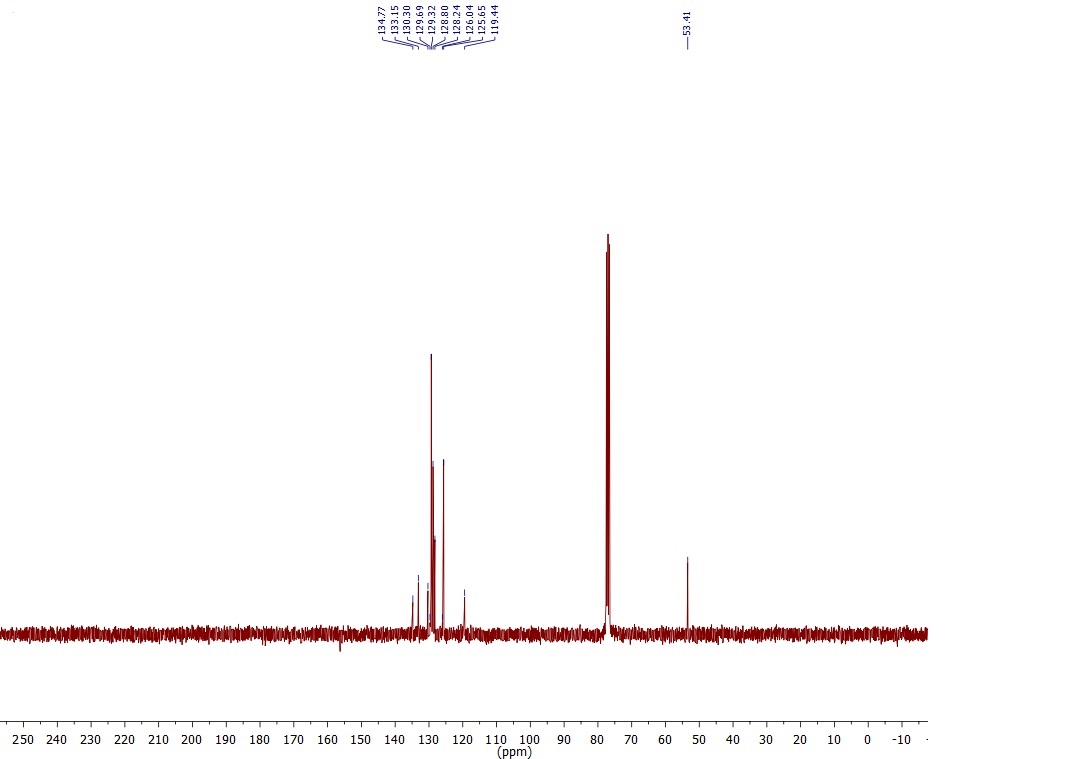
**

**(1-(4-chlorobenzyl)-1H-1,2,3-triazol-4-yl)methanol (compound-3f) 1H NMR:**

**
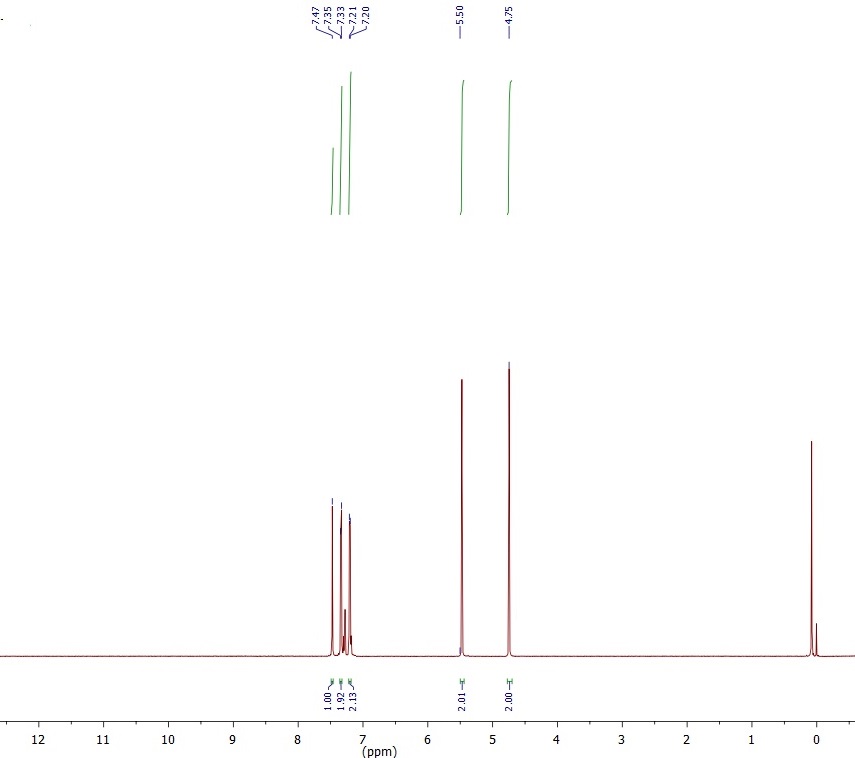
**

**(1-(4-chlorobenzyl)-1H-1,2,3-triazol-4-yl)methanol (compound-3f) 13C NMR:**

**
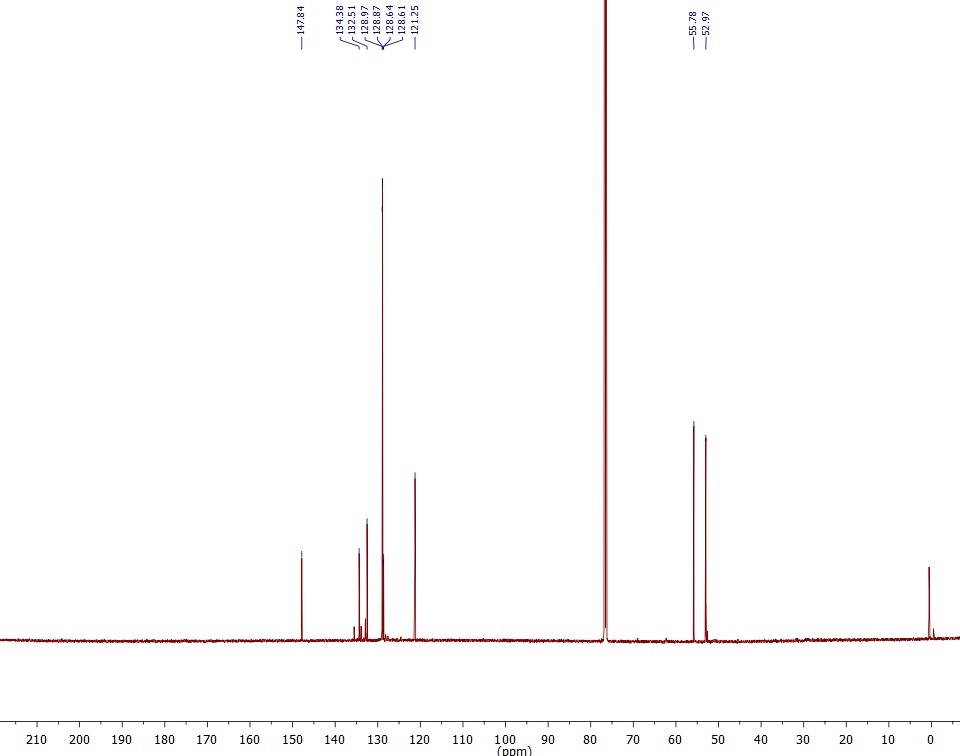
**

**1-(4-methoxybenzyl)-4-phenyl-1H-1,2,3-triazole (compound-3g) 1H NMR:**

**
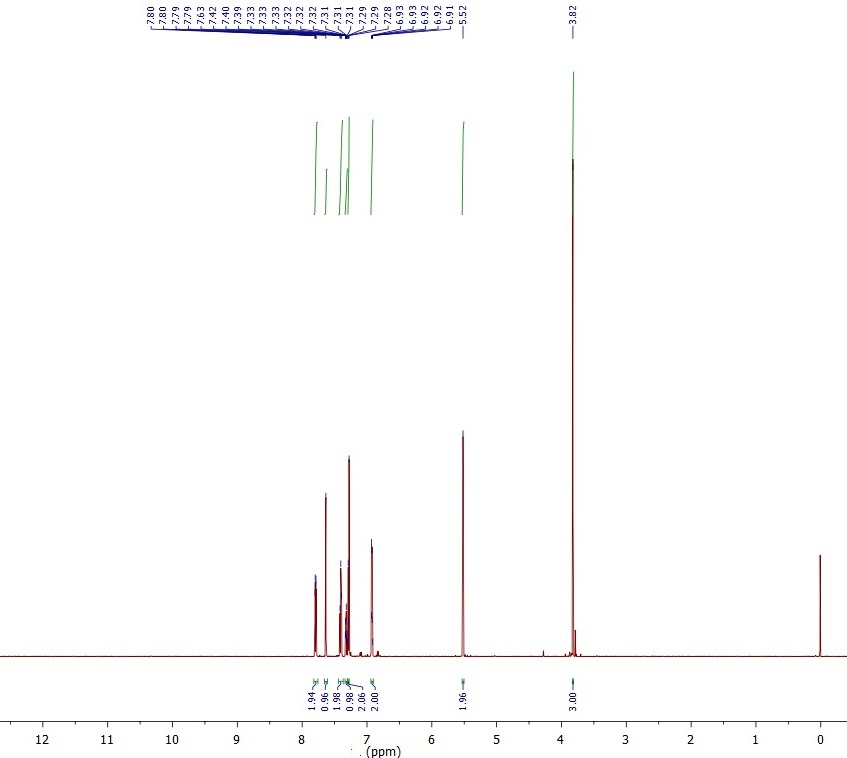
**

**1-(4-methoxybenzyl)-4-phenyl-1H-1,2,3-triazole (compound-3g) 13C NMR:**

**
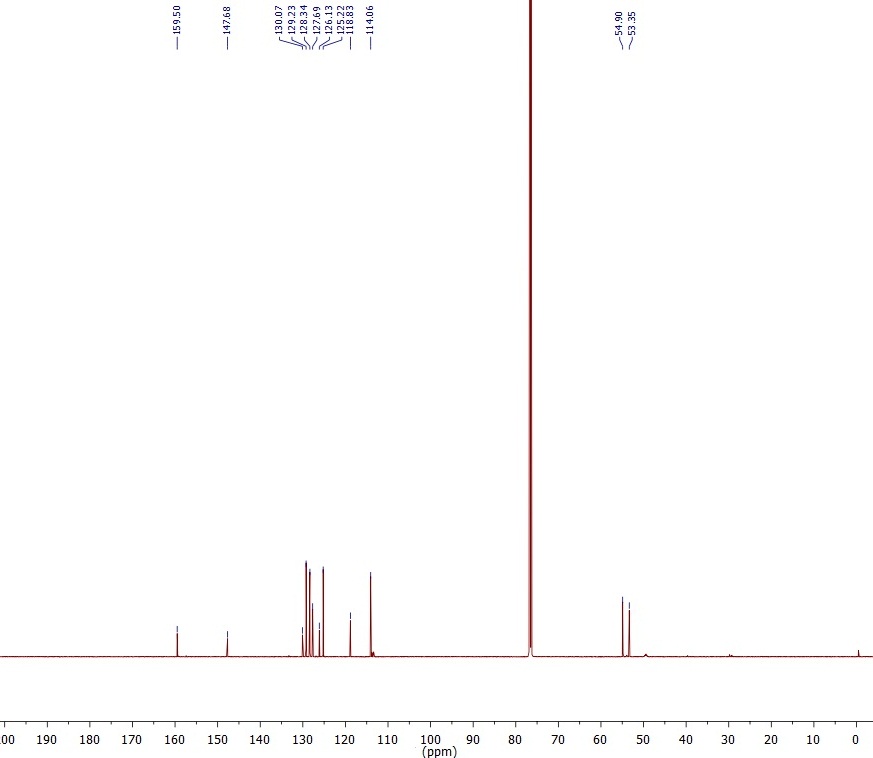
**

**(1-(4-methoxybenzyl)-1H-1,2,3-triazol-4-yl)methanol (compound-3h) 1H NMR:**

**
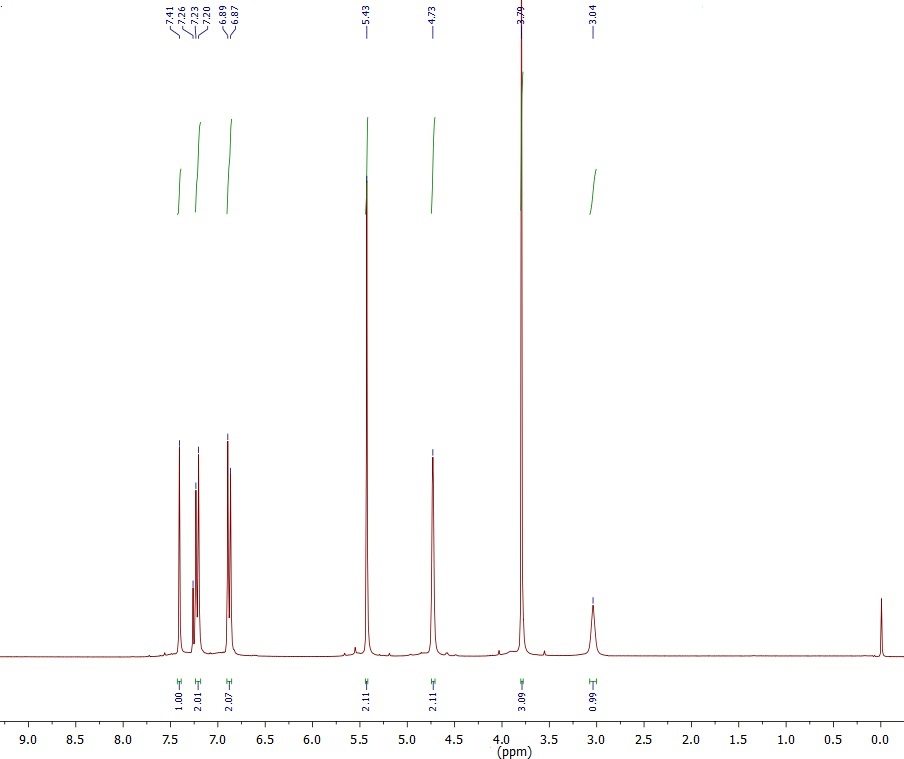
**

**(1-(4-methoxybenzyl)-1H-1,2,3-triazol-4-yl)methanol (compound-3h) 13C NMR:**

**
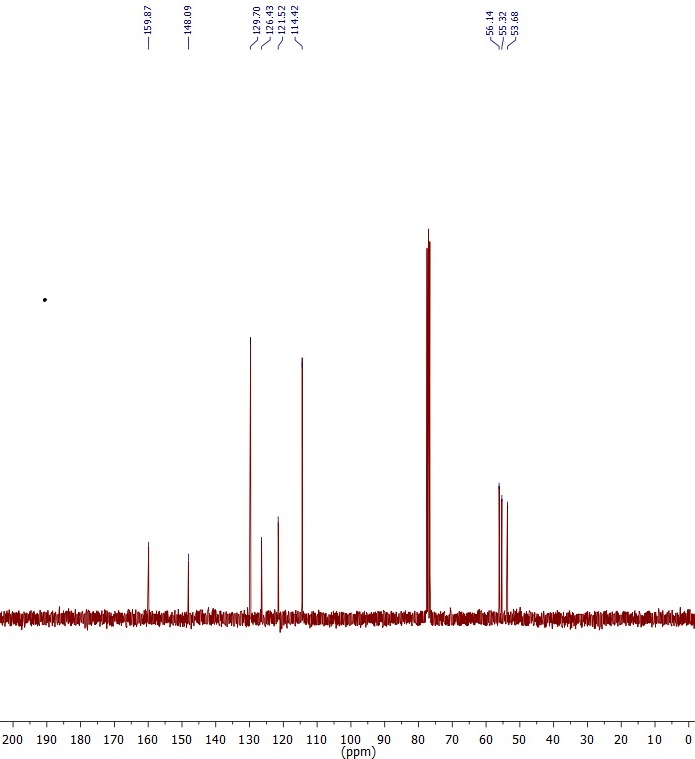
**

**1-(4-nitrobenzyl)-4-(phenoxymethyl)-1H-1,2,3-triazole (compound-3i) 1H NMR:**


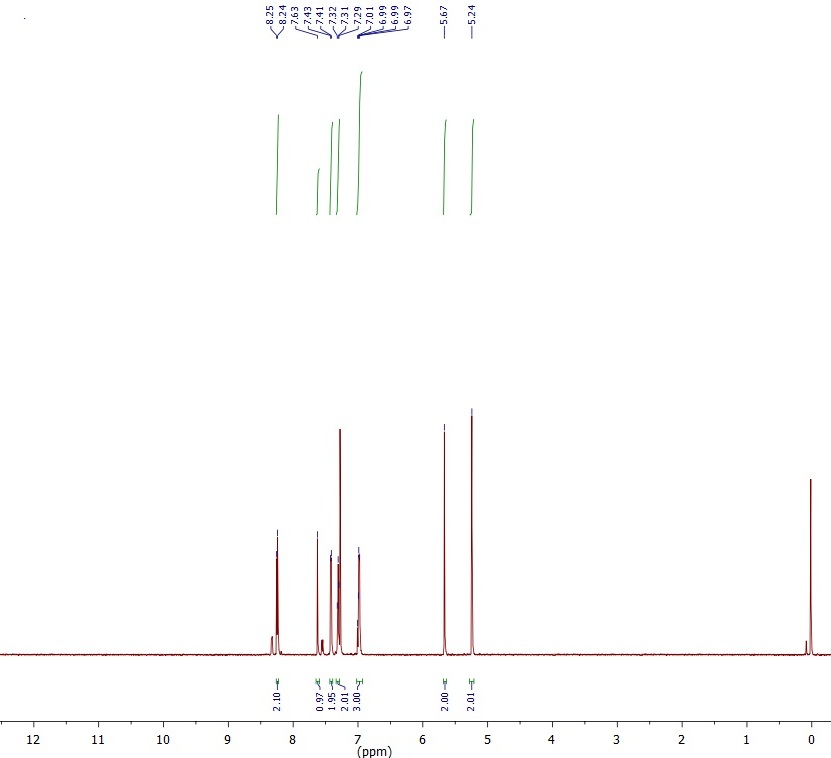


**1-(4-nitrobenzyl)-4-(phenoxymethyl)-1H-1,2,3-triazole (compound-3i) 13C NMR:**

**
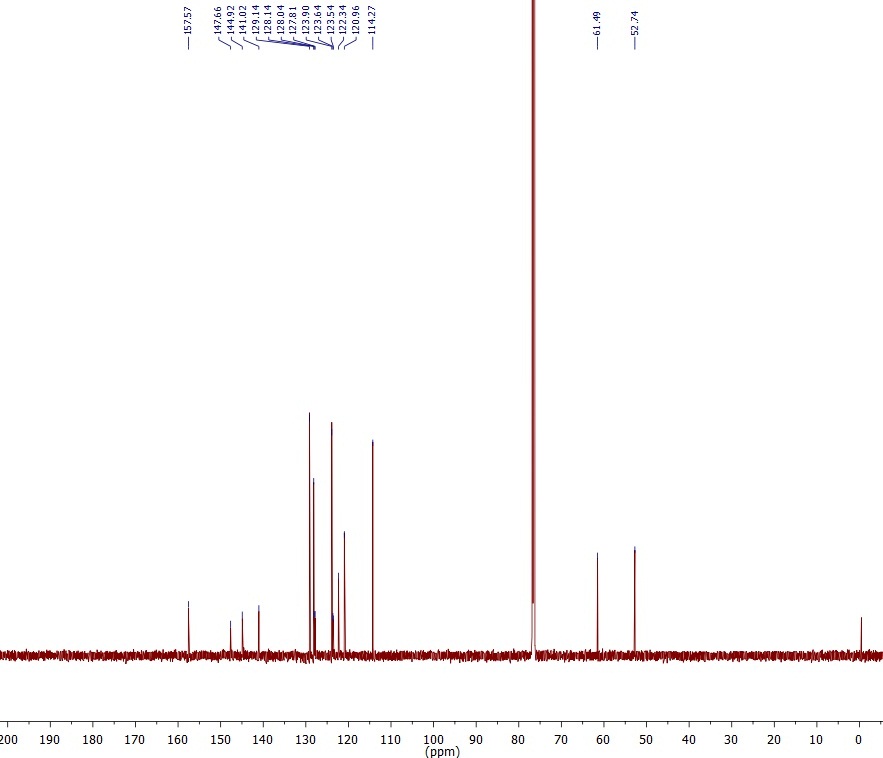
**

**1-(4-methoxybenzyl)-4-(phenoxymethyl)-1H-1,2,3-triazole (compound-3j) 1H NMR:**


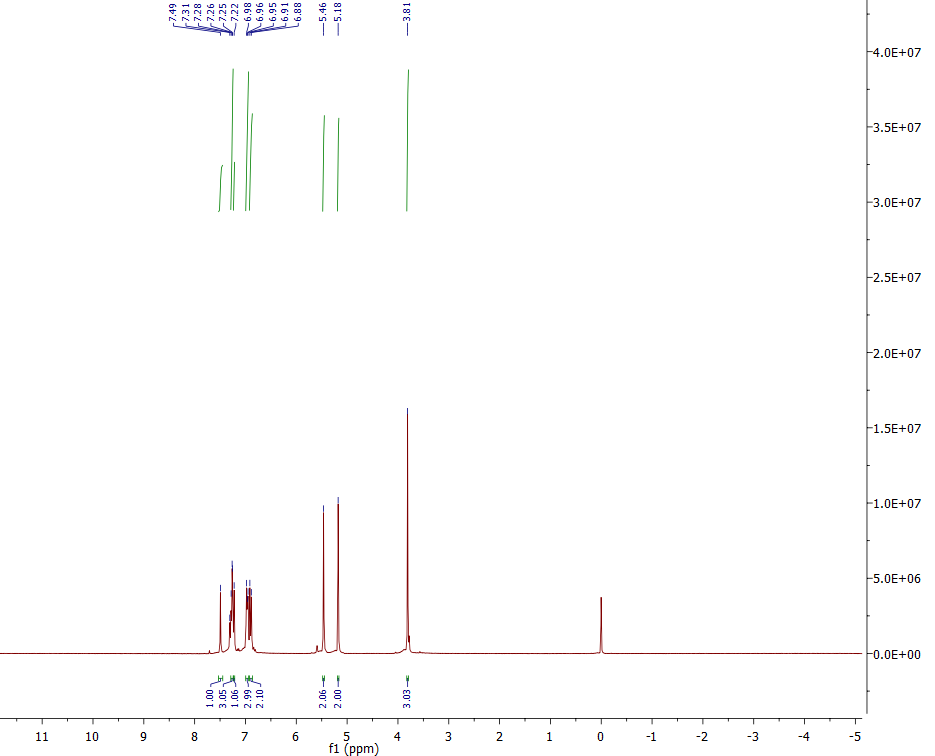


**1-(4-methoxybenzyl)-4-(phenoxymethyl)-1H-1,2,3-triazole (compound-3j) 13C NMR:**


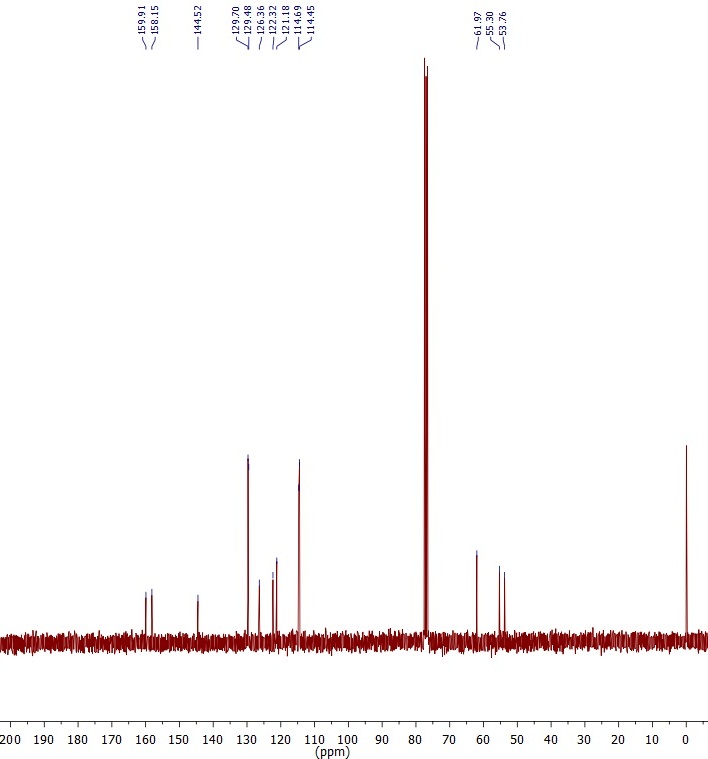


**1-(4-chlorobenzyl)-4-(phenoxymethyl)-1H-1,2,3-triazole (compound-3k) 1H NMR:**

**
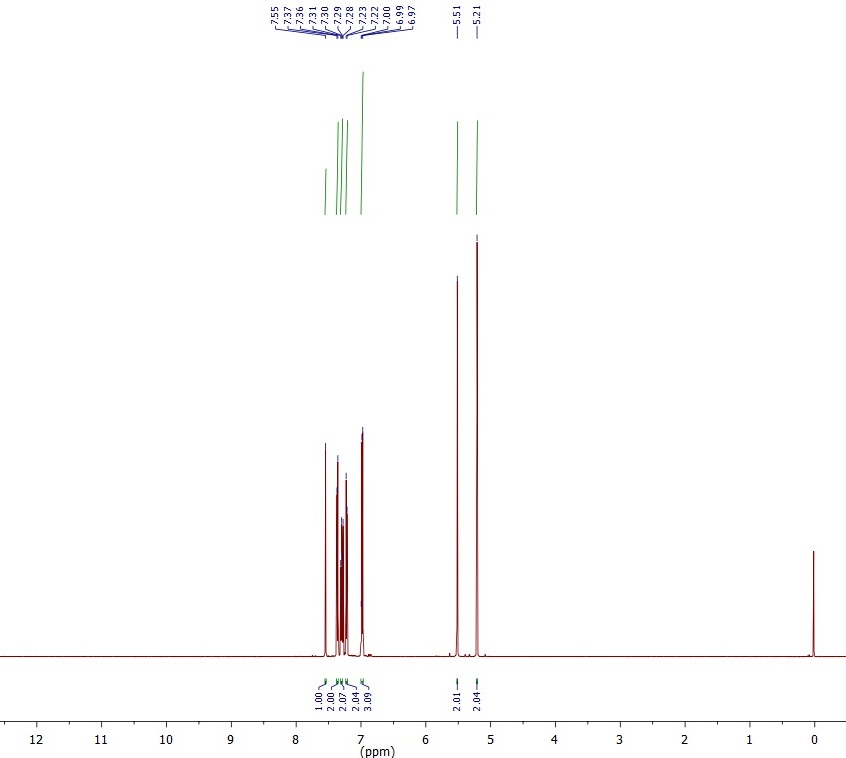
**

**1-(4-chlorobenzyl)-4-(phenoxymethyl)-1H-1,2,3-triazole (compound-3k) 13C NMR:**

**
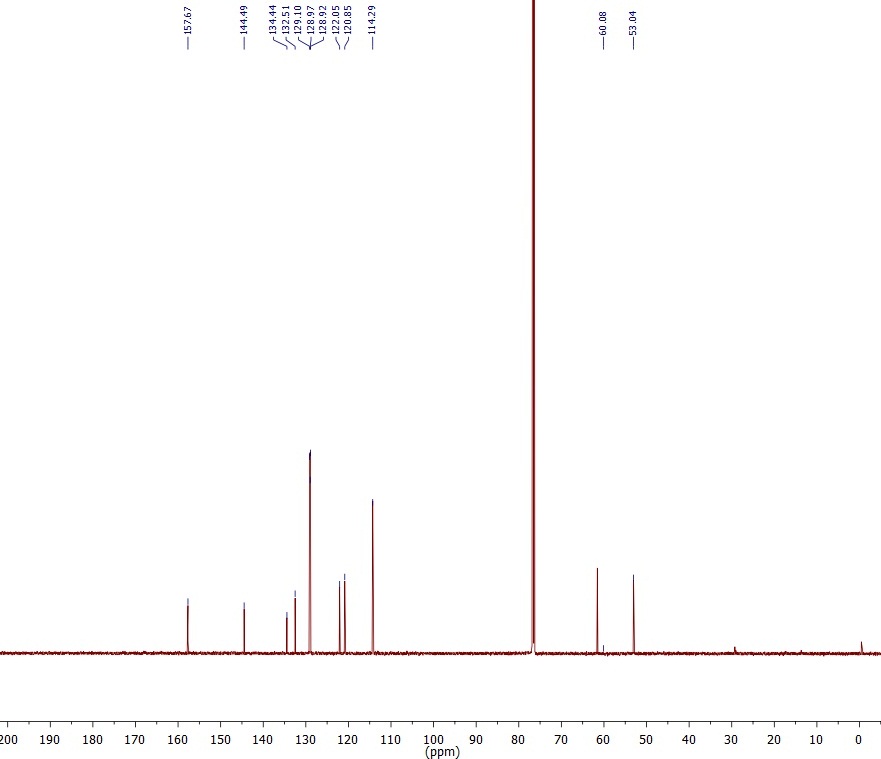
**

**1-benzyl-4-(phenoxymethyl)-1H-1,2,3-triazole (compound-3l) 1H NMR:**

**
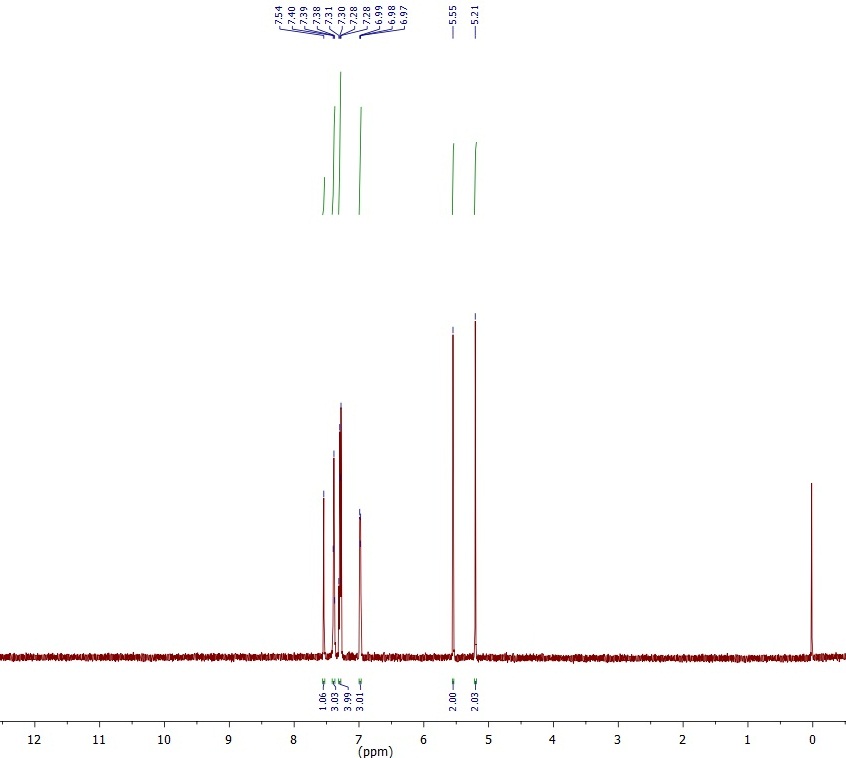
**

**1-benzyl-4-(phenoxymethyl)-1H-1,2,3-triazole (compound-3l) 13C NMR:**

**
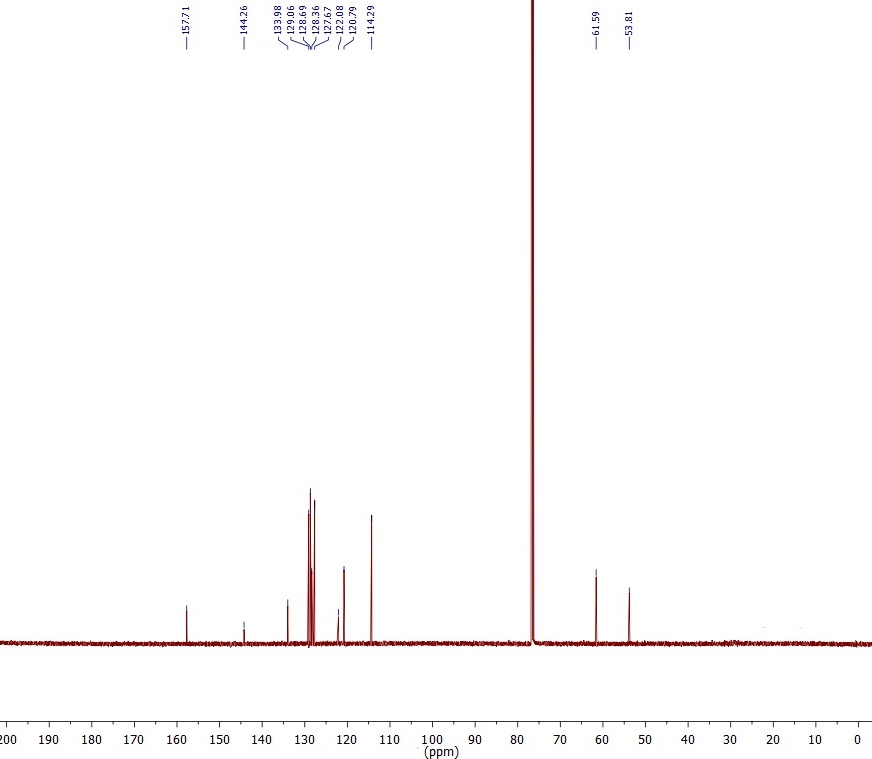
**
